# Applications of Optical Fiber Sensors in Geotechnical Engineering: Laboratory Studies and Field Implementation at the Acropolis of Athens

**DOI:** 10.3390/s25051450

**Published:** 2025-02-27

**Authors:** Elena Kapogianni, Michael Sakellariou

**Affiliations:** Laboratory of Structural Mechanics, National Technical University of Athens, 157 80 Athens, Greece; mgsakel@mail.ntua.gr

**Keywords:** structural health monitoring, optical fiber sensors, centrifuge tests, acceleration sensor, physical modelling, Acropolis of Athens

## Abstract

The current study investigates the feasibility and performance of Fiber Bragg Grating (FBG) optical sensors in geotechnical engineering applications, aiming to demonstrate their broader applicability across different scales, from controlled laboratory experiments to real-world field implementations. More specifically, the research evaluates the sensors’ ability to monitor key parameters—strain, temperature, and acceleration—under diverse loading conditions, including static, dynamic, seismic, and centrifuge loads. Within this framework, laboratory experiments were conducted using the one-degree-of-freedom shaking table at the National Technical University of Athens to assess sensor performance during seismic loading. These tests provided insights into the behavior of geotechnical physical models under earthquake conditions and the reliability of FBG sensors in capturing dynamic responses. Additional testing was performed using the drum centrifuge at ETH Zurich, where physical models experienced gravitational accelerations up to 100 g, including impact loads. The sensors successfully captured the loading conditions, reflecting the anticipated model behavior. In the field, optical fibers were installed on the Perimeter Wall (Circuit Wall) of the Acropolis of Athens to monitor strain, temperature, and acceleration in real-time. Despite the challenges posed by the archaeological site’s constraints, the system gathered data over two years, offering insights into the structural behavior of this historic monument under environmental and loading variations. The Acropolis application serves as a key field example, illustrating the use of these sensors in a complex and historically significant site. Finally, the study details the test setups, sensor types, and data acquisition techniques, while addressing technical challenges and solutions. The results demonstrate the effectiveness of FBG sensors in geotechnical applications and highlight their potential for future projects, emphasizing their value as tools for monitoring structural integrity and advancing geotechnical engineering.

## 1. Introduction

In the modern era, there is an increasing demand for the rapid acquisition of information related to the response of structures under various loading conditions, such as earth pressures, dead and self-weight loadings, as well as those caused by geo-environmental and geophysical hazards, including seismic events [1] (Natural phenomena and hazards typically occur suddenly and with significant intensity, often leading to structural issues or even catastrophic failures. As such, structural monitoring is crucial for hazard assessment, particularly in evaluating the loading conditions and in the early detection of damage or impending failures, which can significantly contribute to enhanced risk management and overall structural safety. By enabling real-time data acquisition, these systems facilitate the development of early warning systems that activate when predefined thresholds are surpassed, thereby enhancing system responsiveness and ensuring timely interventions.

Sensors and monitoring devices are indispensable tools for assessing structural responses to both static and dynamic loadings, applicable across laboratory testing and in the field [2,3,4]. These technologies are utilized in a broad spectrum of projects, ranging from small-scale infrastructure works to large-scale projects, including more recent applications in cultural heritage sites [5]. The selection of an appropriate monitoring strategy is site-specific, depending on factors such as sensor type, installation locations, and the number of required sensors. Moreover, these decisions are influenced by the scale of the project, the characteristics of the site, and its importance. For example, small engineering projects in areas with lower risk levels may demand fewer sensors, while larger, high-priority sites in hazard-prone zones will necessitate a more extensive sensor network.

In civil engineering, optical fiber sensors, such as Brillouin and Bragg Grating technologies, are employed for monitoring structural integrity and measuring key parameters such as strain, stress, and temperature [6,7]. These sensors offer high precision, resilience to mechanical stresses, and the ability to multiplex signals, making them useful in assessing structural health. Their portability allows for easy installation at strategic points for monitoring various loading conditions and assessing distress in critical regions. Brillouin optical fibers are particularly well-suited for large-scale structures like bridges and pipelines, providing long-range strain and temperature monitoring [8,9,10]. In contrast, Bragg Grating optical fibers provide more localized, precise measurements, ideal for monitoring critical points during both laboratory tests and field applications [11,12].

The primary objective of this research is to investigate the feasibility and effectiveness of Fiber Bragg Grating (FBG) optical sensors in geotechnical engineering applications, in both controlled laboratory experiments and real-world field implementations. The FBG optical sensing technique operates based on shifts in the Bragg wavelength, which are influenced by factors such as strain, temperature, and acceleration. These shifts are directly related to changes in the grating’s periodicity and effective refractive index. The calibration of FBG sensors, as provided by the manufacturer, involves the use of specific equations that account for the effects of strain, temperature, and acceleration. For example, wavelength shifts are converted into strain using a calibration equation that incorporates the temperature sensitivity of the sensor and the stress–wavelength relationship. The individual calibration equations for strain, temperature, and acceleration are referenced throughout the following sections.

This study seeks to highlight the significance of FBG sensors in advancing geotechnical monitoring techniques, particularly in their ability to measure critical parameters such as strain, temperature, and acceleration under a wide range of loading conditions, including static, dynamic, seismic, and centrifuge loads. To achieve these objectives, the research explores the application of optical fiber sensors through a series of experimental setups. In the laboratory, experiments were conducted using the one-degree-of-freedom shaking table at the National Technical University of Athens to assess the performance of FBG sensors during seismic events. These tests provided valuable insights into the behavior of geotechnical physical models under earthquake-like conditions, allowing for an evaluation of the reliability and precision of optical fiber sensors in capturing dynamic responses. Additionally, physical models were constructed and tested using the geotechnical drum centrifuge at ETH Zurich. During these tests, optical fiber sensors recorded strain as gravitational accelerations reached up to 100 g, including under impact loading scenarios. The sensors effectively captured the loading conditions, with the recorded data accurately reflecting the expected behavior of the models, demonstrating their robustness and effectiveness under extreme stress conditions. Following the laboratory phase, the study extended to a field application where optical fibers were installed at the Perimeter Wall (Circuit Wall) of the Acropolis of Athens. This installation enabled real-time, remote monitoring of strain, temperature, and acceleration, offering data that were useful for the evaluation of the structural health of this historically significant monument. Despite the inherent challenges posed by working in an archaeological setting, the system proved to be capable of long-term data collection, contributing to the preservation efforts of cultural heritage sites.

Throughout this manuscript, technical challenges, solutions, and data-gathering and interpretation methods are discussed, providing a comprehensive look at the practical implementation of FBG sensors in geotechnical and heritage monitoring. By presenting the results of these investigations, the research underscores the growing potential of optical fiber sensing technology in both geotechnical engineering and the conservation of culturally significant sites, reinforcing the importance of integrating these advanced monitoring tools in future projects for improving the accuracy, safety, and long-term preservation of these structures.

## 2. Structural Monitoring of Scaled Geotechnical Models Under Seismic Loading

Earthquakes and seismic events subject critical infrastructure, such as buildings, slopes, pipelines, bridges, tunnels, and dams, to high stresses and deformations that can compromise their integrity and functionality. Bridges are affected by vertical and horizontal accelerations, often resulting in instability or even failures. Tunnels can experience deformation or cracking, while dams face risks of cracking and displacement, potentially leading to catastrophic flooding. Geotechnical slopes (natural or man-made), including reinforced slopes, are vulnerable to landslides, soil liquefaction, and failures, especially in saturated soils, posing risks to nearby infrastructure. Furthermore, pipelines experience axial and bending stresses from ground motion, fault ruptures, and soil liquefaction, which can cause cracking, joint dislocation, or ruptures.

Assessing the structural response of geotechnical structures requires a combination of advanced tools and methodologies, with physical modelling and structural monitoring being two key approaches. Physical modelling usually involves the use of scaled-down experiments to replicate real-world conditions and study the behavior of geotechnical structures under various loads, including static, dynamic, and seismic loads. These controlled experiments provide valuable insights into stress redistribution, deformation patterns, and failure mechanisms. Furthermore, structural monitoring employs technologies like inclinometers, piezometers, and fiber optic sensors to collect real-time data on strains, displacements, and pore pressures. This continuous monitoring helps track performance over time, detect early signs of instability, and validate numerical/computational models or physical test results. Together, physical modelling and structural monitoring provide a comprehensive framework for understanding and predicting the complex responses of geotechnical systems under varying conditions, contributing to safer and more efficient designs.

**Figure 1:**
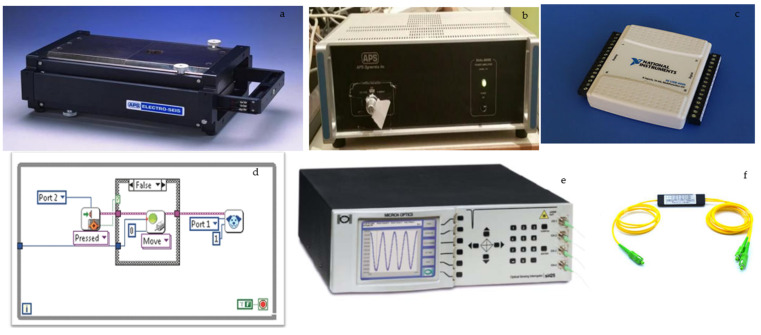
Laboratory equipment: (**a**) single-degree-of-freedom force generator, (**b**) amplifier, (**c**) data acquisition card, (**d**) LabView software, (**e**) interrogator, (**f**) optical fiber sensors.

In the current study, optical fiber sensors were deployed at the laboratory shaking table of the National Technical University of Athens to assess their performance under dynamic and seismic loadings [13]. Specifically, strain and acceleration sensors were attached to various scaled-down physical models to record variations as loading increased. The following section presents the test setup and provides representative recordings. Figure 1 illustrates the laboratory equipment, which includes the following: (a) a single-degree-of-freedom force generator, used to create seismic motion by converting electrical signals into kinetic energy; (b) an amplifier that boosts the low-power supply signal to one capable of directly driving the shaker; (c) a data acquisition card with analog and digital inputs/outputs, facilitating the connection between the computer and the generator, as well as controlling the shaking table’s frequency and magnitude; (d) custom LabView software developed to operate the system; (e) an interrogator for data collection; and (f) the optical fiber sensors used in the experiments.

**Figure 2:**
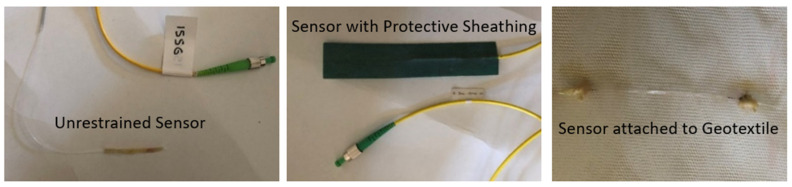
Unrestrained sensor, sensor with protective sheathing, and sensor attached to a geotextile.

Fiber Bragg Grating (FBG) sensors (Figure 2), equipped with different coatings, adhesives, and restraint configurations, were embedded in various geotechnical models constructed within a transparent, watertight box. The following section outlines four representative tests that showcase the performance of these optical fiber sensors in a controlled laboratory environment. Details of each model’s geometric characteristics, as well as the acceleration levels applied via the force generator, are provided, as well as the strain measurements captured by the optical fiber sensors. The wavelength changes recorded by the sensors during the loading events were converted into strain using the following equation:

Δε = (Δλ − Κ_s_ × ΔΤ)/Κ_ε_, (1)

where Δε represents the strain change; Δλ is the wavelength change; Κ_s_ is equal to 11.2 pm/°C, reflecting the temperature sensitivity of the sensors; Κ_ε_ is a factor expressing the stress–wavelength relationship, which equals 1–0.2 pm/με for the sensors used in this study; and ΔΤ is the temperature variation measured during the tests. In this study, no temperature variations occurred due to the short duration of the tests, so the temperature-related term in the equation was disregarded. However, for longer tests, temperature variations should be considered.

This series of tests specifically investigates the seismic behavior and stability of geotechnical slopes, with a strong focus on optical fiber sensor measurements for real-time strain monitoring. The first test, titled “Saturated Sand Slope”, includes a saturated scaled sand slope subjected to seismic loading. The second test, “Lower Acceleration Response”, explores how reduced seismic accelerations influence the strain response in a similar slope model, highlighting the sensitivity of optical fiber measurements. The third test, “Pipe Reinforcement Effects”, investigates the impact of pipe reinforcement on slope stability and deformation under seismic conditions, with optical fiber sensors monitoring strain distribution. The fourth test, “Reinforced Vertical Slope”, assesses the effectiveness of geotextile reinforcement in a vertical slope model, using optical fiber sensors to monitor strain behavior and failure under seismic loading. These tests collectively aim to enhance the understanding of slope behavior under dynamic forces and failure mechanisms, with particular emphasis on the capabilities of optical fiber sensors in providing real-time data.

As mentioned during the shaking table tests, loading conditions were controlled using a single-degree-of-freedom force generator, which produced seismic motion with adjustable frequency and amplitude managed by custom LabView software and a data acquisition card. Furthermore, seismic loads were applied incrementally and monitored in real-time using additional integrated equipment, the Quake-Catcher Network-QCN [14]. This sensor, incorporated into the experimental setup, offered the advantage of measuring acceleration in three axial directions, ensuring precise load application while enhancing the accuracy of the collected data. Additionally, to ensure the accuracy of the optical fiber laboratory measurements, each individual experiment was conducted multiple times to verify the consistency of the recorded data and to eliminate potential errors during the data acquisition process. This repetition, combined with calibration, helped identify and address discrepancies. It should also be noted that the reliability of the measurements is also achieved by the interphase between the fiber and the soil when it comes to the scaled physical models. In other words, strains recorded by the optical fibers represent soil strains.

**Figure 3:**
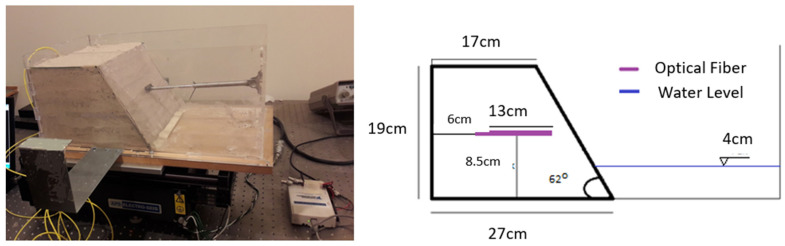
Saturated sand slope: model and geometrical characteristics.

**Figure 4:**
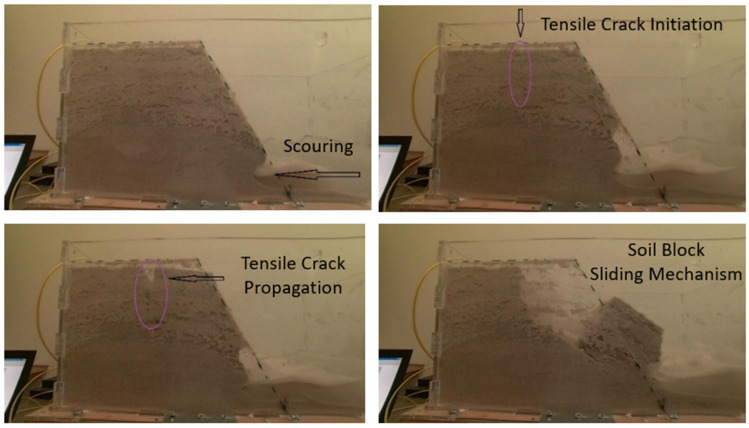
Saturated sand slope: model behavior and resulting failure mechanism.

During the “Saturated Sand Slope” test, a scaled sand slope model was constructed within the transparent, watertight box mounted on the earthquake simulator (Figure 3). An optical fiber sensor was embedded in the slope model, attached to a 13 cm-long geotextile, positioned 8.5 cm above the base and oriented as illustrated in Figure 3. This specific location was selected because the failure mechanism was anticipated to occur nearby, making it an ideal spot to monitor and capture data with the optical fiber sensor. A small, predetermined amount of water was incorporated during construction, with a water level set at 6 cm to examine failure mechanisms in the presence of water. The maximum applied acceleration during the test reached 3.8 m/s². A digital camera, positioned on the earthquake simulator platform, captured the entire failure sequence (Figure 4). As anticipated, erosion/scouring occurred at the slope’s base due to water movement. Additionally, a diagonal tensile crack developed at the top of the model, signaling strain localization, a typical pre-failure behavior in granular slopes. The crack propagated, leading to the sliding of a significant soil block and the complete failure of the model.

The failure of the scaled sand slope model during this test can be attributed to the combined effects of dynamic loading, water-induced instability, and the inherent properties of granular materials. The dynamic acceleration of 3.8 m/s² from the earthquake simulator generated cyclic shear stresses, leading to particle rearrangement and a reduction in shear strength. The presence of water at a 6 cm level introduced hydrodynamic pressure, reducing the effective stress and frictional resistance within the slope. This condition facilitated erosion at the slope base due to water movement, undermining stability and initiating failure. Simultaneously, the cyclic loading caused strain localization, manifesting as a diagonal tensile crack near the slope’s top, a common precursor to failure in granular slopes. The propagation of this crack formed a failure plane, resulting in the sliding of a significant soil block and the complete collapse of the model.

**Figure 5:**
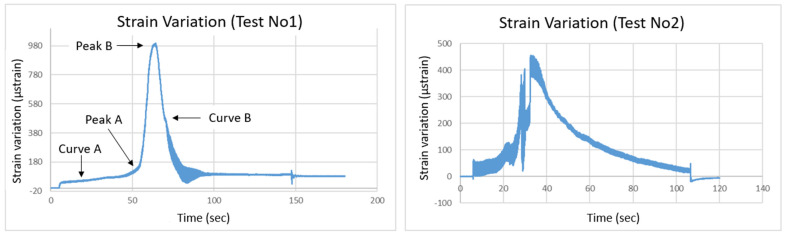
Saturated sand slope (**left**) and lower acceleration response (**right**): strain variation recorded by the optical fiber sensors.

In Figure 5 (left), strain recordings obtained by the optical fiber sensor are displayed as the slope model was subjected to seismic loading. As expected, the strain increased during the applied seismic load, with the highest strain values occurring just before the slope’s failure. Notably, in the approximately 50-s period leading up to failure, strain levels remained relatively low, reaching up to 180 μstrain. However, as the crack at the top of the slope widened, strain levels sharply increased, peaking at 980 μstrain during the slope’s collapse. Specifically, Curve A in the diagram shows a small gradual strain increase during seismic loading, culminating in a maximum point (Peak A) which corresponds to the initiation of the crack. As the crack expanded further, a second, much higher peak (Peak B) was recorded just before the large soil mass slid, signaling total failure. This could indicate either a transition from elastic to plastic deformation, or the strain increase may result from the rising stress at the crack tip. Another possible explanation is that the optical fiber was experiencing tension due to the failure, which caused the soil to separate into two distinct sections. After the collapse, the strain values decreased significantly, as shown in Curve B, reflecting the failure of the soil mass, which no longer responded to the seismic loading. The model’s behavior and failure mechanism aligned with expectations based on the literature and were reflected by the optical fiber sensor strain recordings.

The second model (“Lower Acceleration Response” test) with similar geometric characteristics was subjected to lower acceleration levels, up to 2.8 m/s^2^, and the strain variations recorded by the optical fiber sensor are shown in Figure 5 (right). The lower strain levels observed in this test are directly related to the reduced seismic acceleration of 2.8 m/s^2^ compared to 3.8 m/s^2^ in the previous test (i.e., “Saturated Sand Slope” test). The sensor measurements clearly show a decrease in maximum strain values, reflecting the lower stress and deformation in the model due to the reduced acceleration. This behavior is consistent with geotechnical principles, where smaller seismic forces result in lower strain responses. The optical fiber data effectively demonstrate how variations in seismic loading influence the mechanical behavior of the slope, highlighting the sensor’s ability to capture such differences under different seismic conditions.

**Figure 6:**
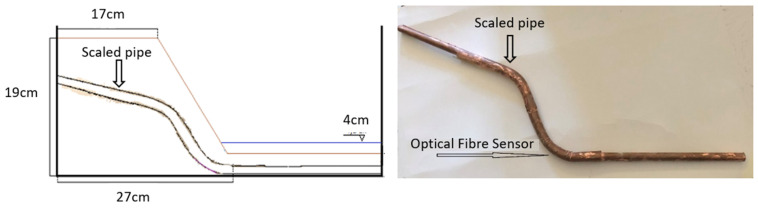
Pipe reinforcement effects: side view of the model (**left**) and the scaled pipe with optical fiber sensor placement (**right**).

**Figure 7:**
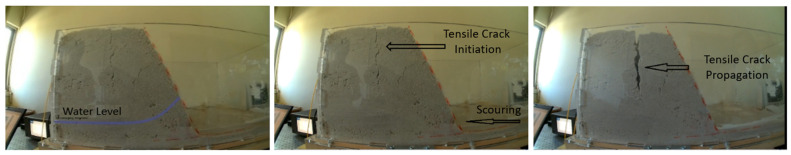
Pipe reinforcement effects: model structural response to applied loading, including scour at the slope base and shear initiation at the existing tensile crack.

**Figure 8:**
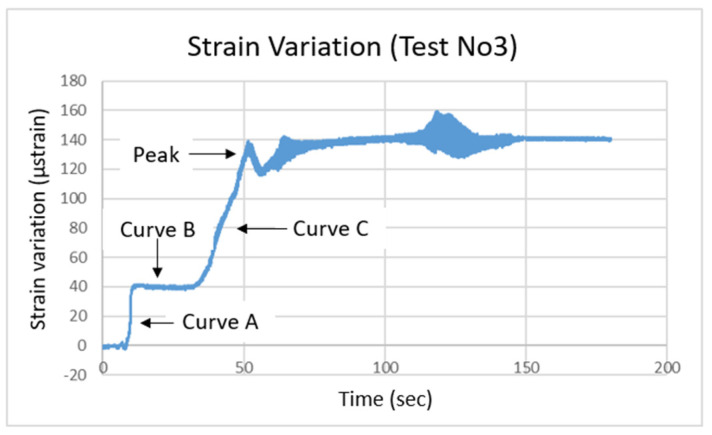
Pipe reinforcement effects: strain variation recorded by the optical fiber sensor.

During the “Pipe Reinforcement Effects” test, an optical fiber sensor was attached to a scaled pipe placed at its estimated critical point, where higher stress concentrations were anticipated. This setup was designed to simulate the use of pipes as reinforcement elements within the slope, where they serve to support the soil, redistribute pressure, and reduce strain. The purpose of this test was to evaluate how the presence of the reinforcing pipe influences the stress distribution and deformation behavior of the slope. More specifically, a maximum seismic loading of 4 m/s^2^ was applied, and the water level was set at 4 cm. Figure 6 provides a side view of the slope model, with the embedded scaled pipe; Figure 7 shows the model’s deformation during seismic loading; and Figure 8 displays the corresponding strain recordings. As observed in Figure 7, seismic loading caused visible scouring at the base of the slope, as well as shear initiation at the pre-existing tensile crack at the top of the slope. Figure 8 illustrates the escalation of strains during seismic loading. Specifically, Curve A depicts the initial increase in recorded strain as seismic loading progressed. Curve B shows a brief stabilization period before transitioning to Curve C, where a sharp increase in strain was observed, ultimately reaching its peak just before failure. It can also be noted that strains on the pipe observed during this test were significantly smaller than strains observed in the previous two tests (i.e., Saturated Sand Slope and Lower Acceleration Response), even though the acceleration imposed was higher. This is both logical and expected, as the presence of the scaled pipe acts as reinforcement, increasing the slope’s overall stability. Despite this, the failure mechanism mirrored that of the earlier tests. Finally, post-test inspection identified visible deformation on the pipe at the sensor’s location, justifying the optical fiber’s placement at this specific location.

**Figure 9:**
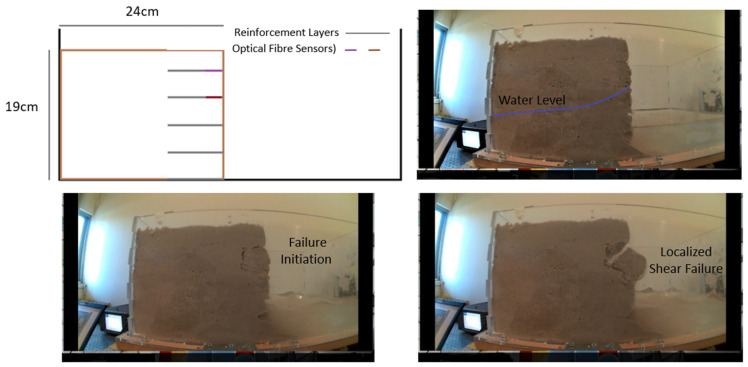
Reinforced vertical slope: cross-section and failure mechanism.

**Figure 10:**
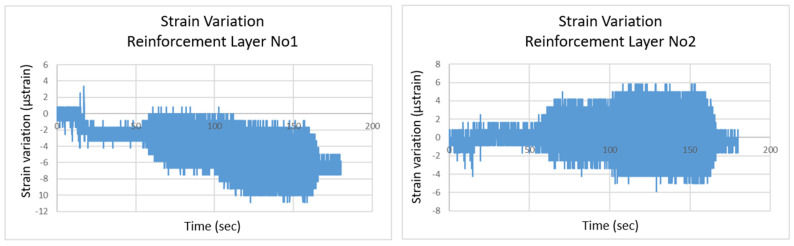
Reinforced vertical slope: strain variation recorded by two optical fiber sensors.

The “Reinforced Vertical Slope” test involved a reinforced vertical slope model, with two optical fiber sensors embedded within two geotextile reinforcement layers, with a maximum applied acceleration of 4 m/s^2^. Figure 9 illustrates the reinforced model, providing both a side view of its structure and the corresponding failure mechanism. The structural response of this model differed significantly from the unreinforced slopes tested earlier. As shown in Figure 10, the failure was a localized shear failure rather than a complete or catastrophic collapse, which is typical for reinforced slopes. It was primarily confined to the slope’s face, and the strain levels recorded by the optical fiber sensors were significantly lower than in previous tests, as anticipated. This test underscores the critical role of reinforcement layers in geotechnical slopes. The reinforcement allowed for the successful construction of a vertical slope model, and despite the high acceleration, strain levels remained minimal. Additionally, the failure was localized to specific areas of the model, with no overall collapse. These results highlight the effectiveness of reinforcement in maintaining slope stability under seismic loading, as was also confirmed by the optical fiber sensor recordings.

## 3. Optical Fiber Sensors and Enhanced Gravity-Centrifuge Loading

Optical fiber sensors were deployed at the drum geotechnical centrifuge of ETH Zurich to enable strain recordings during centrifuge load increases and impact loading scenarios, with the setup of the ETH drum centrifuge further described by Springman et al. [15]. Geotechnical centrifuges are widely used to experimentally investigate scaled-down geotechnical structures by applying a gravitational field that is “*n*” times greater than Earth’s gravity. This scaling is crucial, as the dominant forces affecting soil structure behavior are gravity-driven, making small-scale testing under normal gravity insufficient [16,17,18]. The optical fiber sensors were affixed to the scaled physical slope models, with minimal coating materials to prevent interference with the model behavior, ensuring as accurate strain variation measurements as possible. The primary objective of this application was to assess the performance of the sensors within the centrifuge environment and to study the behavior of the scaled models under these loading conditions.

**Figure 11:**
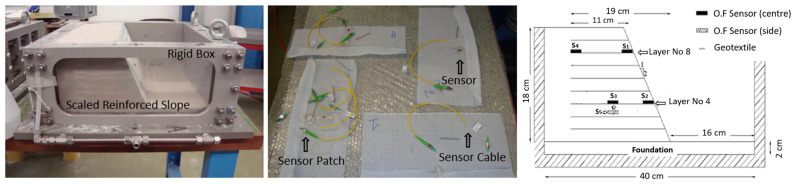
Scaled reinforced slope model (**left**), reinforcement layers incorporating optical fiber sensors (**middle**), and model cross-section (**right**).

Several mechanically-stabilized earth (MSE) scaled-down slope models were built with the same geometry inside a strong box, incorporating uniformly spaced reinforcement materials to simulate reinforced soil slopes, as can be seen in Figure 11 [19]. Textile-embedded optical fiber strain sensors were strategically attached on the reinforcement layers to monitor strain development during the loading scenarios. A transparent, colorless glue that cures when exposed to ultraviolet light was used to attach the sensors to the scaled reinforcement sheets. The sensors were embedded using a fast-curing two-component adhesive, consisting of a liquid and a powder, which created fixed boundaries to prevent the sensors from sliding. The soil material used for the models was a fine-grained uniform sand from the west coast of Australia [20].

**Figure 12:**
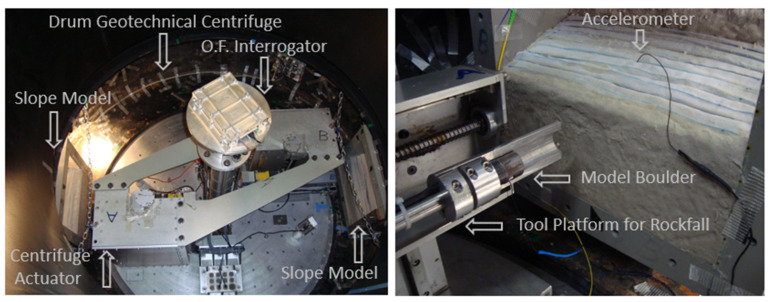
Test setup (**left**) and reinforced slope in the centrifuge (**right**).

Each optical fiber had an individual/unique initial wavelength, and wavelength variations were recorded as g levels increased. A portable optical fiber interrogator, positioned along the centrifuge’s axis inside a rigid box to protect it from high g-forces, was used for data collection (Figure 12). The sensors were connected in series through a splicing device, and the optical fiber data acquisition was made possible using SmartSoft V3.2.0 software. This configuration enabled simultaneous strain measurements every second, due to the distinct initial wavelengths of the sensors. Impact loading was simulated using a steel block to represent a boulder falling onto the slope models. Impact loading was investigated, and a model block made of steel was used to simulate a boulder falling on the slope models. The boulder was attached to a magnet on the tool platform, which was rotating together with the drum. The purpose of the electro-magnet was to hold the block during the test to release it at a desirable moment. A guiding tube was used along the fall path of the block to ensure orthogonal contact between the block and the surface of the slope and also to keep the block in the n-g field to achieve high energy levels [21]. Vibrations generated by the impact were measured by two Brüel and Kjær accelerometers (HBK Nærum, Denmark). The maximum acceleration recorded for the model boulder during impact reached 9.7 m/s^2^. A digital camera was positioned alongside the models to capture images throughout the loading events for PIV analysis. Initially developed for experimental fluid mechanics [22], PIV has been applied to study soil behavior in the physical modeling of geotechnical structures [23]. In this study, GeoPIV [24] was utilized.

The strain (Δε) was determined using Equation (1), as previously described, considering the sensor’s stress–wavelength relationship. Since the tests were brief and temperature variations were negligible, the temperature-related term was omitted.

**Figure 13:**
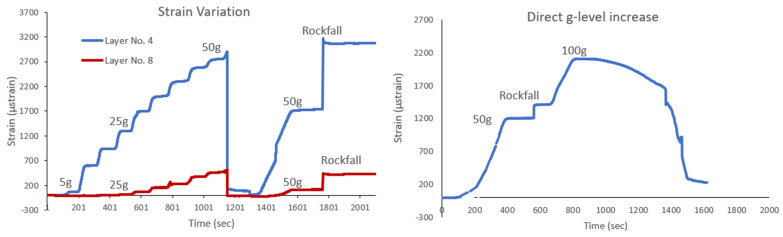
Strains captured during Test No. 1 (**left**) and strains captured during Test No. 2 (**right**).

Wavelength recordings were captured every one second, and various loading scenarios were applied. The first loading scenario involved increasing the g level by 5 g every 2 min, reaching a maximum of 50 g. This was followed by an unloading phase and a subsequent direct increase in the g level to 50 g. Afterward, the model boulder was released, initiating the rockfall event. The strains recorded, as shown in Figure 13 (left), are logical, expected, and consistent with the corresponding loading scheme. The strain measurements, obtained from two optical fiber sensors positioned at different locations within the slope model, are shown for this test. Sensor 8 was placed at a higher elevation within the model, while Sensor 4 was positioned at a lower elevation. Specifically, at a g level of 50 g, Sensor 4 measured a maximum strain variation of Δε_4max_ = 2840 μstrain, while Sensor 8 recorded a maximum strain variation of Δε_8max_ = 1440 μstrain. The larger strain variation observed in Sensor 4 can be attributed to the increased overburden pressure at that depth, which amplifies the strain response. The direct reapplication of the g level up to 50 g was also detected by the optical fiber sensors. After reaching this loading level, the model boulder was released, and its impact on the top of the slope was captured by the sensors. As expected, the sudden increase in strain due to the impact was significantly higher for the sensor located in the upper layers of the slope, given its closer proximity to the boulder.

Figure 13 (right) presents recordings from a second test conducted on a similar geotechnical model. In this case, the loading scheme was different, with the g level increasing directly from 1 g to 50 g. The maximum strain recorded was Δε_4max_ = 1200 μstrain, which is significantly lower than the strains observed in the previous test. Impact loading was also applied at the 50 g level, and again, the recorded strains were smaller. The maximum g level reached was 100 g, and the optical fiber sensors successfully captured wavelength—and thus strain—variations at this high acceleration. The direct reapplication of the g level to 50 g and the subsequent rockfall impact caused a more pronounced strain increase in the upper layers, where Sensor 8 was positioned closer to the boulder. During Test 2, the smaller maximum strain recorded is attributable to the different loading scheme, where the g level was increased directly from 1 g to 50 g, resulting in lower strain responses due to reduced acceleration. The results from both tests demonstrate the optical fiber sensors’ capacity to measure strain under varying loading conditions, confirming their effectiveness in capturing geotechnical behavior under dynamic and high acceleration environments.

The study demonstrated that optical fiber sensors are well-suited for small-scale constructions and that minimizing sensor duplication reduces their influence on scaled models. Sensors can be connected in series via splicing, enabling simultaneous measurements at multiple locations while providing reliable and comparable data. The monitoring system developed proved to be robust enough to withstand extreme gravitational accelerations of up to 100 g. The results from the various tests aligned with theoretical expectations in both form and magnitude. The recorded measurements reflected the applied loading conditions, and the sensors effectively captured the impact loading scenario within the models.

**Figure 14:**
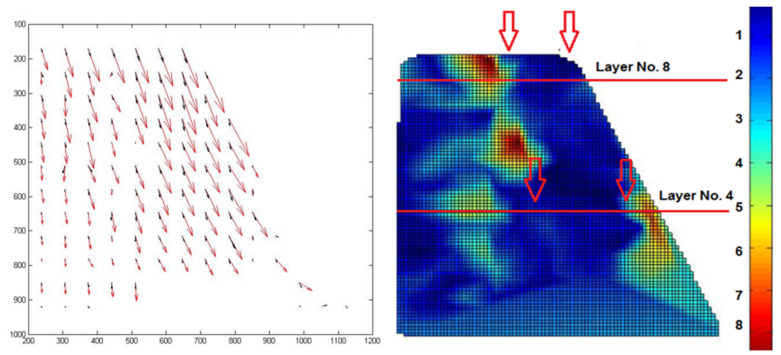
Flow vectors of soil grains, with vectors colored black representing 1 g and vectors colored red indicating 50 g (**left**). Normalized strain values calculated via GeoPIV and locations of the FBG sensors (**right**).

As mentioned previously, within the framework of this experimental study, Particle Image Velocimetry (PIV) analysis was performed to observe failure mechanisms and correlate the findings with optical fiber recordings [25]. GeoPIV [24] was employed to assess strains and failure mechanisms in the scaled slope models as the g level increased. GeoPIV is a MATLAB module that adapts PIV techniques for geotechnical testing, allowing for the collection of displacement data from sequences of digital images captured during the experiments. Figure 14 (left) illustrates the flow vectors of soil grains, with vectors colored black representing 1 g and vectors colored red indicating 50 g. Figure 14 (right) displays strains calculated through GeoPIV alongside the locations of the optical fiber sensors integrated into the physical models. In GeoPIV, the vertical scale represents normalized strain values rather than absolute strain. To convert these normalized values into microstrain, a scaling factor based on a reference measurement can be used. For example, if a GeoPIV value of 6 corresponds to the maximum optical fiber measurement equal to 2.840 μstrain, then each unit represents approximately 470 μstrain. This analysis underscores the potential for combining different complementary methods for enhanced structural monitoring during physical modelling with optical fibers or other monitoring techniques.

**Figure 15:**
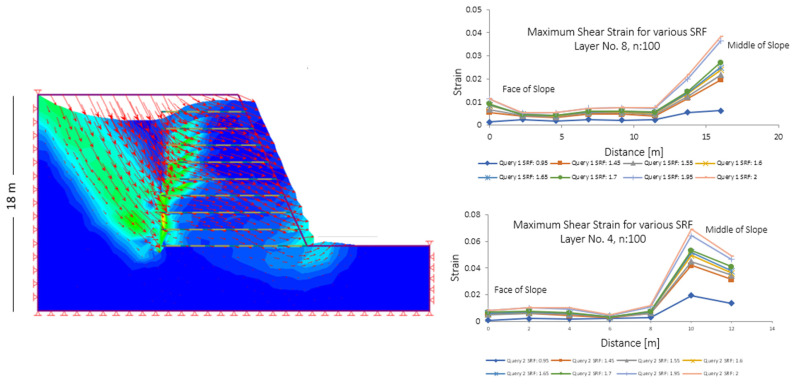
Numerical model of full-scale slope (**left**) and maximum shear strains in Layers No. 8 and No. 4, at different SRF levels (**right**), using FEM.

For the scaled-down models studied in the centrifuge, corresponding computational/numerical models were developed using the Finite Element Method (FEM), aiming to examine their expected theoretical behavior. The analysis utilized the Finite Element Stress Analysis Method with the Strength Reduction Factor (SRF) approach, enabling the determination of shear strain values. The maximum shear strains for various SRF levels are presented for specific reinforcement layers. Specifically, layers No. 8 and No. 4 were selected, located near the crest/face and mid-slope, respectively. The corresponding results are illustrated in Figure 15 for a scale factor of n = 100, representing a slope height of 18 m according to the scaling laws. As observed, for higher SRF values, the shear moments increase, while as the SRF approaches unity, corresponding to the actual state of a slope, the values decrease. These results are logical, consistent with expectations, and align with findings from experimental investigations and instrumented measurements.

In summary, for this experimental setup, each optical fiber sensor was connected in series and had a unique initial wavelength, while the portable interrogator, shielded from high g-forces, recorded wavelength variations. These variations were converted into strain measurements using a calibrated equation (Equation (1)) that accounted for potential temperature effects, although no temperature fluctuations were expected during the centrifuge tests. Loading conditions during the tests were systematically controlled by incrementally increasing the g levels from 5 g to 50 g, with strain variations captured every second. The setup also simulated impact loading by releasing a model boulder onto the slope, with a guiding tube ensuring orthogonal contact. Challenges in ensuring uniformity across repeated tests arose from slight sensor positioning variations and setup inconsistencies; however, repeated testing and advanced analysis methods, including Particle Image Velocimetry (PIV) and numerical/ computational model comparisons [26], validated the reliability of the measurements. This comprehensive approach ensured the sensors’ effectiveness in monitoring strain variations under centrifuge loading conditions.

## 4. Optical Fiber Sensors at the Acropolis of Athens

The current section presents the use of optical fibers for structural monitoring at the Acropolis of Athens. Cultural heritage sites, such as the Acropolis, are of paramount importance to both national and international heritage, yet they face significant risks, with the potential for irreversible damages. Assessing the structural risks at these sites is a complex and critical task, as they are exposed to various natural and human-made hazards throughout their lifecycle. Effective structural monitoring plays a key role in evaluating these risks and is vital for ensuring the safety of visitors.

The Acropolis, a UNESCO World Heritage site, symbolizes classical civilization and stands atop a rocky Hill about 150 m above sea level and 70 m above the city of Athens. The Hill has a trapezoidal shape, measuring roughly 350 m in length and 150 m in width, and forms part of the Lycabettus–Tourkovounia–Acropolis ridge complex [27]. Its geology consists of limestone over Athenian schist, with the site surrounded by fortification walls built over 2500 years ago. The Acropolis, despite its historical and cultural importance, has endured considerable damage from both natural hazards and human actions, including wars, invasions, pollution, and seismic activity, all of which have progressively increased the risk of structural degradation. Notably, studies have recorded specific seismic events impacting the site, such as those occurring in 1705 and 1805 in Athens, as well as in 1837 in Troezen [28]. Among the standing monuments on the Acropolis Hill, such as the Parthenon, the Propylea, and the Erechtheion, the Perimeter Wall serves a pure geotechnical purpose, since it functions as a typical gravity wall, retaining the backfill that forms the plateau of the Acropolis.

**Figure 16:**
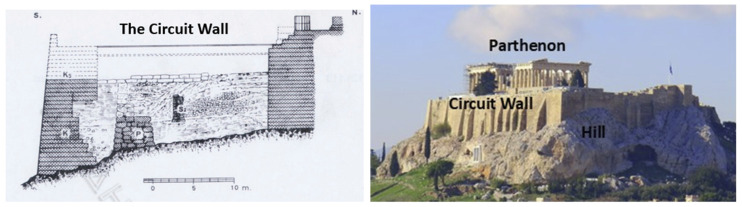
Cross-section of the southern part of the Circuit Wall (**left**) [29]. A panoramic view of the Acropolis Hill, the Circuit Wall, and the Parthenon from the southeast (**right**).

The Circuit Wall of the Acropolis Hill is an ancient masonry retaining wall primarily composed of irregular mixed courses of marble and small stones added during subsequent repairs. It spans approximately 800 m in length and varies in height from 5 to 18 m. Figure 16 (left) shows a cross-section of the southern part of the Circuit Wall (left) [29], and Figure 16 (right) shows a panoramic view of the Acropolis Hill, the Circuit Wall, and the Parthenon from the southeast.

Aiming to investigate the structural integrity of the Acropolis Circuit Wall and Hill, a monitoring scheme with FBG optical fiber sensors has been developed. This monitoring system consists of eight active optical fiber strain sensors, with two sensors attached to each of the four smart rods (one on the inner side and one on the outer side, labeled as IN and OUT, respectively), along with two temperature optical fiber sensors attached on another smart rod and one optical fiber acceleration sensor attached directly on the Circuit Wall, all transmitting real-time data remotely. The smart rods were attached using stainless steel plates anchored to the substrate, allowing them to be easily detached, if necessary, since no adhesive was applied. Optical signal transmission was facilitated by an optical sensing interrogator, and the sensors were connected and spliced in the field in both series and parallel configurations. Regarding the array of optical fiber in both series and parallel configurations, this setup was necessary due to the limited number of available channels on the interrogator, which were only four to accommodate a total of eleven sensors (i.e., eight strain sensors, two temperature sensors, and one acceleration sensor). To address this, some sensors were connected in series, and these series connections led to four optical fiber cables, which were routed in parallel to the four channels. The distinct initial wavelengths of the sensors helped prevent signal overlap, allowing each sensor to be individually identified.

**Figure 17:**
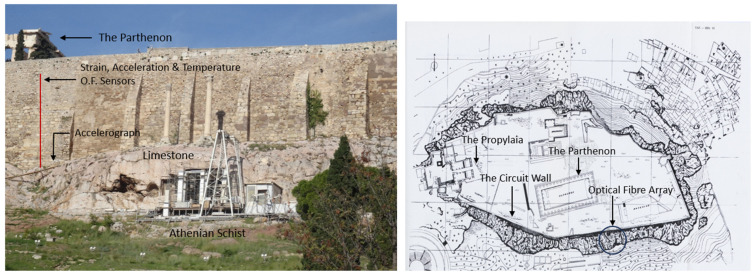
Locations of the installed optical fiber sensors on the South Wall (**left**) and the plan view of Acropolis Hill (**right**) [29].

Figure 17 highlights the locations of the installed smart rods and the FBG acceleration sensor on the South Wall and at the Acropolis Hill. This specific location of the Circuit Wall was chosen for the optical fiber array installation for several key reasons, which are as follows: (a) The South Wall in this area reaches one of its greatest heights (~18 m) along the Circuit Wall and contains a significant volume of backfill material. (b) Visible cracks have been detected in this section of the South Wall, indicating structural vulnerability. (c) The selected area is positioned near two pre-existing accelerographs, which form part of the accelerographic network on Acropolis Hill [30].

**Figure 18:**
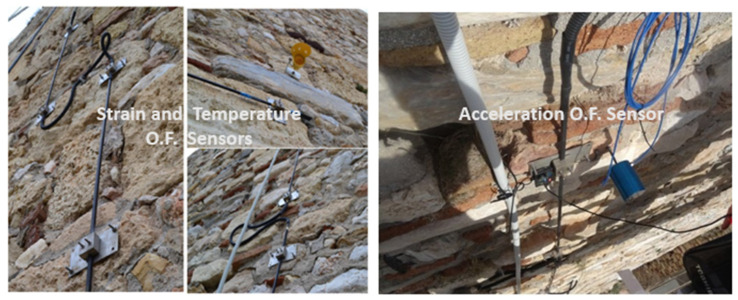
Strain and temperature FBG sensors on the Wall, including anchoring plates (**left**) and acceleration FBG sensor (**right**).

**Figure 19:**
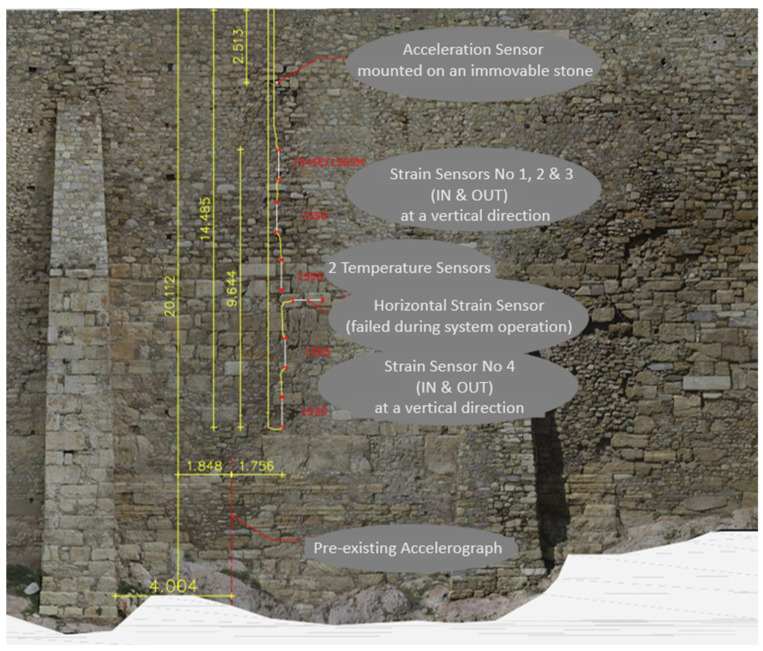
Configuration of Fiber Bragg Grating sensors, arranged in series and parallel on the South Circuit Wall.

Figure 18 displays the strain, temperature, and acceleration sensors installed on the Wall, including the anchoring steel plates. More specifically, the smart rods, to which the strain and temperature optical fiber sensors were attached, are shown on the left of Figure 18, while the optical fiber single-axis acceleration sensor, positioned perpendicular to the Wall, is shown on the right. Figure 19 illustrates the locations of the sensors on the Acropolis Circuit Wall. This setup complements an existing monitoring system, with 10 high-quality broadband accelerographs positioned strategically across the Acropolis Hill. These devices provide continuous recordings, with 24-bit digitizers transmitting real-time data for ongoing evaluation.

To convert the recorded wavelength variation to strain values, the aforementioned Equation (1) was used. An example of this calculation at the Acropolis Circuit Wall is as follows: given λ_1_ = 1566.247 nm = 1,566,247 pm and the reference value λ_0_ = 1566.064 nm = 1,566,064 pm, the wavelength shift is Δλ_1_ =183 pm. Using Equation (1), the corresponding strain is calculated as Δε_1_ = 183 pm/(1.2 pm/μstrain) = 152.5 μstrain. For the optical fiber single-axis acceleration sensor, the relationship between wavelength shifts and acceleration is determined by the sensor’s sensitivity to dynamic forces. The primary objective is to measure the wavelength shift, Δλ, resulting from applied accelerations. Changes in acceleration induce a corresponding shift in the Bragg wavelength, which is directly proportional to the applied acceleration. This relationship is expressed by the following equation:

Δλ_i_ = S⋅a_i_, (2)

where Δλ_i_ represents the wavelength shift (in pm) from the reference value (average or dataset), S is the sensor’s sensitivity (S = 75 pm/g in this study), and a_i_ is the acceleration (in units of g or m/s^2^). In order to convert the wavelength variation to acceleration, the final equation was used for the current study:

a_i_ = (Δλ_i_/75)∙g. (3)

An example of the calculations is as follows: for λ_1_ = 1561.063 nm and the reference value of λ_0_ = 1561.062 nm, it is calculated that Δλ_1_ = 0.001 nm = 1 pm and a_1_= Δλ_1_/S = 0.0133 g. The specifications and sensitivity of the single-axis acceleration sensor used in this study are provided in Table 1.

To ensure precise measurements in environments with varying temperatures, it is crucial to account for temperature effects, which can induce wavelength shifts similar to those caused by strain or acceleration. Temperature variations affect the Bragg wavelength through the two following primary mechanisms: thermal expansion of the optical fiber, which alters the grating spacing, and changes in the refractive index of the material. These temperature-induced shifts are unrelated to strain or acceleration and must be decoupled to ensure accurate measurements.

For strain sensors, a temperature sensor placed near the strain sensors allows the temperature-induced shift to be quantified and subtracted from the total wavelength shift. This isolates the strain-induced wavelength shift, providing accurate strain measurements. Similarly, in single-axis acceleration sensors, the same temperature-induced effects must be accounted for using the sensor’s temperature coefficient (in nm/°C). By incorporating this coefficient, the temperature effects can be corrected, isolating the acceleration-induced wavelength shift for precise acceleration measurement.

The total observed wavelength shift, Δλ_total_, is the combined result of both strain and temperature effects, and is expressed by the following relationship:

Δλ_total_ = Δλ_strain_ +Δλ_temperature_, (4)

where Δλ_total_ is the measured total wavelength shift, Δλ_strain_ is the component due to strain, and Δλ_temperature_ is the component due to temperature changes. By subtracting Δλ_temperature_ from Δλ_total_, the strain-induced wavelength shift can be accurately isolated, ensuring precise measurement of the strain.

**Figure 20:**
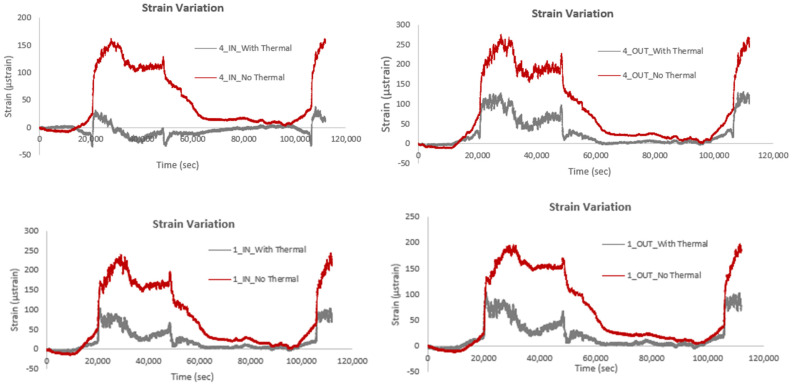
Comparison of strain variation with and without thermal compensation.

The following figures illustrate the strain, acceleration, and temperature recordings from the Circuit Wall of the Acropolis of Athens. An initial analysis investigates the effect of temperature on strain measurements. Specifically, Figure 20 compares strain data with and without thermal compensation for sensors No. 1 and No. 4, positioned at both the locations labeled IN and OUT. The subfigures demonstrate that sensors installed at different heights on the wall (e.g., sensor 4 and sensor 1) exhibit subtle differences in their responses to temperature variations. Similarly, sensors located at the same position but on the inner (IN) and outer (OUT) sides of the smart rod show varying sensitivities to temperature effects. Overall, the findings show that strain values are higher when temperature effects are not compensated for, which is as expected. The increased strain values when temperature is not taken into consideration can be attributed to the sensitivity of optical fiber sensors to temperature fluctuations. As mentioned previously, in optical fiber strain sensors, such as those based on Fiber Bragg Grating, temperature changes can induce shifts in the fiber’s refractive index and grating spacing, which are misinterpreted as strain. Without compensation for these temperature-induced shifts, the total observed wavelength shift includes both strain and thermal effects, leading to inflated strain measurements. Implementing thermal compensation allows for the isolation of strain-induced wavelength shifts, ensuring that the measurements accurately reflect the mechanical deformation of the structure.

**Figure 21:**
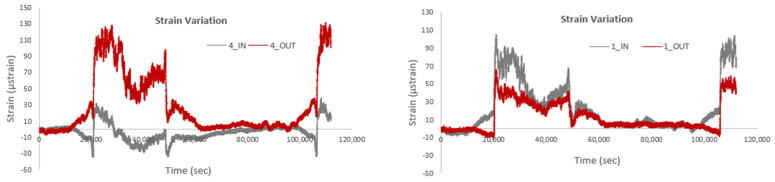
Comparison of strain variation at the IN and OUT positions for the same smart rods.

Figure 21 depicts a comparison of strain variation at the IN and OUT positions for the same smart rods. It is observed that for sensor No. 1, the variation between the inner and outer sensors is minimal, whereas for sensor No. 4, the corresponding variation is significantly larger for the sensor located at the OUT position. It should also be noted that the data presented show measurements following thermal compensation. The difference in strain variation between sensors No. 1 and No. 4 could be attributed to several factors, such as position and environmental exposure, structural factors, sensor sensibility, boundary conditions, and load distribution. The overall study shows that No. 4 is positioned in an area more exposed to environmental stressors or thermal gradients compared to sensor No 1, leading to higher strain differences. Furthermore, variations in material properties or localized stress concentrations in the rod could result in more pronounced strain differences at specific positions. Finally, sensor No. 4 might exhibit a higher sensitivity to strain compared to sensor No. 1, amplifying the observed variation.

**Figure 22:**
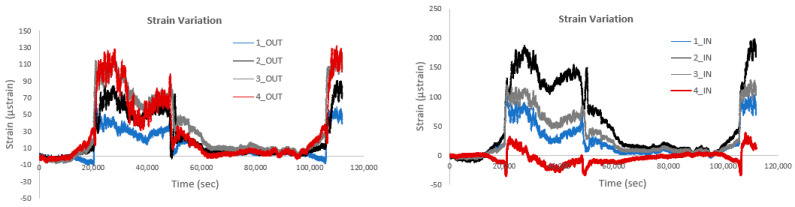
Strain variation of four sensors at both IN and OUT positions, with thermal compensation.

Figure 22 illustrates the strain variation for four sensors placed on the four smart rods at both the IN and OUT positions, with thermal compensation applied. As observed, for the sensors at the OUT positions, the highest strains are recorded by sensor No. 4, while the lowest strains are noted by sensor No. 1. Conversely, for the sensors at the IN positions, sensor No. 2 exhibits the highest strains, while sensor No. 4 shows the lowest. This pattern aligns with previous observations, where sensor No. 4 at the OUT position experiences the greatest strain variation. The differences can be explained by the varying exposure of the sensors to temperature gradients, structural properties, or external forces at different positions. Sensor No. 4, located at the OUT position, is likely subjected to more significant environmental or mechanical influences, resulting in higher strain, whereas sensor No. 1 at the same position is less affected. Meanwhile, sensor No. 2 at the IN position may be more sensitive to temperature-induced strain or other localized effects, contributing to the higher readings observed.

Strain patterns exhibit a clear temporal variation, with higher values typically recorded between morning and noon. This increase is primarily associated with restoration activities at the Acropolis, including the operation of heavy machinery, crane movements, and the transport of large monument components. The presence of a substantial number of visitors during this period further contributes to the observed strain levels. Additionally, rising temperatures in the late morning hours lead to thermal expansion effects on the Wall and backfill. As expected, this trend gradually reverses during the afternoon and evening.

**Figure 23:**
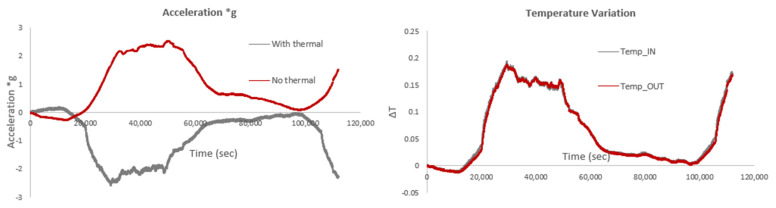
Acceleration levels recorded by the single-axis acceleration sensor with and without thermal compensation using the initial wavelength value (**left**) and temperature variation (**right**).

The following figures present the acceleration levels recorded by the single-axis acceleration optical fiber sensor over the same time period. Figure 23 (left) examines the impact of temperature on the measurements, revealing a significant effect. As observed, the acceleration values (a·g) recorded show opposite directions—positive without thermal compensation and negative with compensation—indicating that the results would have been substantially different in the absence of thermal compensation. Figure 23 (right) illustrates the temperature variation recorded by the two temperature sensors (IN and OUT positions, respectively), which coincide. When thermal compensation is not applied, temperature-induced changes—such as expansion or contraction of the sensor materials, or shifts in the sensor’s characteristics (e.g., refractive index, grating spacing in fiber sensors)—can introduce false readings. These temperature effects can cause the acceleration values to appear in the opposite direction (positive without compensation and negative with compensation), as thermal expansion or contraction may alter the sensor’s measurements in a way that mimics or counteracts the actual acceleration. It is also worth noting that the accelerometers capture all dynamic movements, from environmental “noise” to seismic events when they occur. Since no earthquakes occurred during this recording period, the data collected and shown in Figure 23 reflect ambient vibrations.

**Figure 24:**
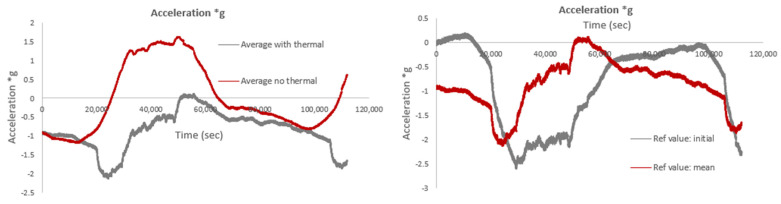
Acceleration levels, both with and without thermal compensation, using the mean wavelength as the reference value (**left**), and a comparison of results with the initial wavelength as the reference value versus those with the mean wavelength as the reference value (**right**).

The following figures compare acceleration levels (a·g) with and without thermal compensation, using the average/mean wavelength measured during this time period as the reference wavelength, instead of the initial measurement used in the previous figures. In this case, the difference between the acceleration values with and without thermal compensation is smaller. Additionally, Figure 24 compares accelerations calculated using the mean wavelength as the reference to those calculated using the initial wavelength, including thermal compensation. As observed, the peak acceleration values are lower when the mean wavelength is used; however, the overall pattern of acceleration variation (i.e., increases/decreases and higher/lower values) remains similar in both cases.

The smaller difference in acceleration values with and without thermal compensation, when using the mean wavelength as the reference, occurs because the mean wavelength likely reflects a more stable, averaged condition over the measurement period, reducing the impact of short-term temperature fluctuations. When the mean wavelength is used, the sensor’s response to temperature-induced shifts is effectively averaged out, which minimizes the discrepancies between the compensated and non-compensated readings. This leads to more consistent acceleration values, regardless of the thermal compensation. In contrast, using the initial wavelength as the reference may result in larger variations because it represents a snapshot at a specific time, which could be more sensitive to temperature changes or other environmental factors. The peak acceleration values are lower with the mean wavelength due to the smoothing effect of averaging, but the overall trend in acceleration variation remains similar, as the underlying acceleration dynamics have not changed; only the reference point for measurement has shifted.

**Figure 25:**
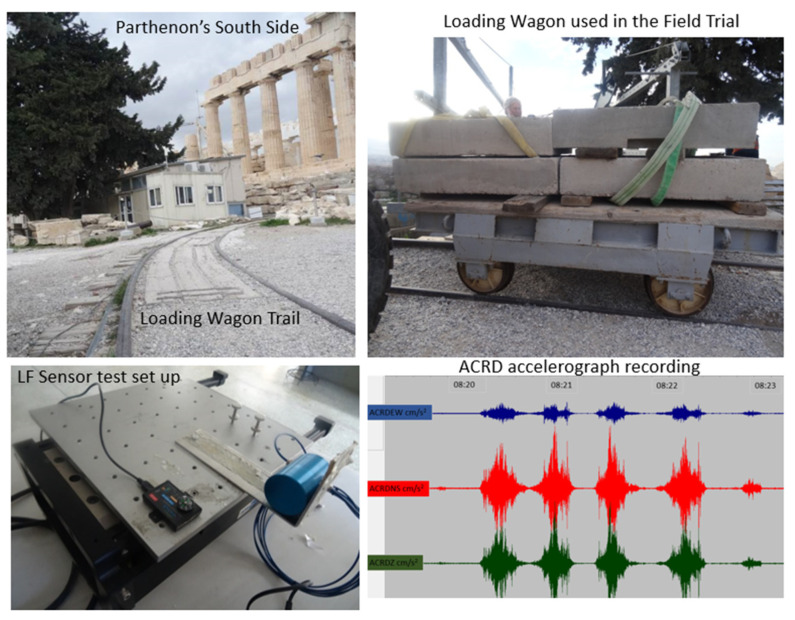
Calibration procedure for the monitoring system at the Acropolis.

In order to facilitate calibration of the installed monitoring system, the following approach was employed. Initially, the complete equipment setup was studied at the laboratory of the National Technical University of Athens, specifically utilizing the single-degree-of-freedom seismic simulator to replicate seismic conditions. To further validate the instruments, a field trial loading was performed. This involved moving a loaded wagon along the Acropolis Hill, approximately 5 m from the Wall, while simultaneously capturing data from the LF acceleration sensor, the strain optical fiber sensors, and the existing accelerograph network. These tests yielded valuable insights into the performance of the installed network, and these findings are detailed by [31]. The nearby ACRD accelerograph recorded the passage of the loaded wagon (Figure 25), capturing acceleration in the following three directions: E–W (blue), N–S (red), and vertical (green). The highest acceleration was observed in the N–S components, which is expected due to their orientation relative to the South Wall and the dominance of vertical vibrations in high-frequency, near-field motion. The bottom-right subfigure provides a qualitative assessment of the vibrations, as it indicates the relative magnitude of acceleration in each direction, rather than presenting exact numerical values.

A general note regarding the installation of the monitoring system at the Acropolis of Athens is that it presented several challenges due to the historic and constrained environment. Securing a reliable electrical power supply was difficult, given the need to ensure continuous operation of the equipment. This issue was addressed by installing a power system with a sufficiently large battery (12VDC, 45Ah), charger, and a computer system to manage data collection. The installation process was further complicated by the 18 m height of the Circuit Wall outside the Acropolis plateau, requiring precise equipment placement and careful handling to avoid any damage to the monument. Additionally, strict limitations on drilling to prevent harm to the historical structure necessitated an alternative approach. The smart rods were fixed without adhesion, using stainless steel plates anchored to the wall, allowing for detachment if needed, without causing permanent damage. Extreme weather conditions on the Acropolis Hill, including cold, windy winters and scorching hot summers, also posed a challenge. The complexity of the system installation, which involved connecting sensors in both series and parallel configurations, required precise fieldwork. Moreover, transmitting sensor data over long distances was difficult due to signal degradation, which was mitigated by using a WiFi antenna for real-time data transmission. The system was designed to be autonomous in terms of power supply, communication, and data collection. These experiences offer valuable insights that can inform future field applications in similar settings, highlighting the importance of careful planning, minimizing the impact on historic sites, and developing robust systems capable of withstanding environmental challenges.

## 5. Discussion

This study demonstrated the effectiveness of optical fiber sensors in geotechnical applications, both in controlled laboratory settings and real-world field conditions. It highlighted the sensors’ capability to provide real-time measurements of strain, temperature, and acceleration responses, offering valuable insights into soil–structure interactions and the behavior of geotechnical structures. More specifically, during the seismic loading tests, scaled soil models were subjected to seismic forces, and optical fiber sensors were strategically placed to monitor strain variations induced by the shaking. The results confirmed the sensors’ high performance, as they successfully measured strain variations with minimal interference. Moreover, the failure mechanisms observed during the tests were in very good agreement with the strain data captured by the sensors, validating their ability to detect and track the structural response to dynamic loading. These findings underline the importance of optical fiber sensors in advancing the understanding of soil–structure interactions and their potential for providing reliable data during seismic events.

The centrifuge tests, on the other hand, involved scaled models of reinforced soil slopes subjected to high gravitational forces to simulate full-scale conditions. The placement of optical fiber sensors on the reinforcement layers of these models allowed for the continuous monitoring of strain variations during both steady increases in gravitational loading and dynamic impact events, such as a falling boulder. The ability of the sensors to operate effectively under extreme conditions, such as accelerations up to 100 g, was demonstrated. The recorded strain data were consistent with the observed failure mechanisms, further confirming that the optical fiber sensors reflected the response of the models under varying loads.

Both the shaking table and centrifuge tests emphasized the versatility of optical fiber sensors in monitoring geotechnical models under a wide range of loading conditions. The integration of complementary techniques, such as Particle Image Velocimetry (PIV) and numerical/computational modeling of the centrifuge tests, enriched the analysis by providing a more detailed understanding of the mechanisms behind failure zones. This combined approach can help to verify the strain data and provide a more comprehensive interpretation of the observed phenomena.

Beyond laboratory-based studies, this research introduced a novel application of Fiber Bragg Grating (FBG) sensors for monitoring the Acropolis of Athens, a UNESCO World Heritage Site of immense cultural and historical significance. This research marks, to the authors’ knowledge, the first use of FBG sensors at such an archaeological site, opening up new possibilities for the preservation and monitoring of cultural heritage monuments. The integration of FBG sensors to measure strain, temperature, and acceleration offers a comprehensive approach to assessing the structural integrity of the Acropolis, particularly the Circuit Wall, a vital geotechnical component of the site. The sensors allowed for real-time, remote data acquisition, enabling the continuous tracking of strain, temperature, and acceleration across various points of the wall. These data may provide valuable insights into the performance of the structure, allowing for early detection of potential issues, such as localized stresses or damage, before they could pose a significant threat to the wall’s structural integrity.

One of the key challenges in monitoring sites like the Acropolis is accounting for temperature-induced errors that can affect the accuracy of the measurements. In this study, thermal compensation techniques were implemented to account for temperature effects during strain and acceleration measurements. This is very important in environments like the Acropolis, where temperature variations are significant and can lead to misleading data without proper calibration. The results from the Acropolis monitoring revealed how environmental and structural factors interact to influence the strain distribution across the Circuit Wall. Variations in strain at different sensor locations highlighted the complexity of the wall’s behavior, underscoring the need for a more detailed approach to structural monitoring.

Regarding historical sites like the Acropolis Circuit Wall, FBG sensors can play an important role in modern conservation strategies. These sensors can provide non-invasive, real-time monitoring of critical structural parameters, offering data to assess the health and stability of such culturally significant monuments. By embedding FBG sensors within the structure, it is possible to detect early signs of stress, strain, and environmental changes that could affect the integrity of the site. This can lead to targeted conservation measures, enhancing preservation efforts without compromising the integrity of the monument.

## 6. Conclusions

FBG sensors offer several advantages and can be effectively utilized across a broad spectrum of geotechnical projects, from laboratory testing and physical modeling to large-scale applications. They enable distributed sensing over long distances and allow for the measurement of multiple parameters (e.g., strain, temperature, and acceleration) along a single fiber by splicing sensors with different initial wavelengths. Furthermore, their compact size and lightweight design facilitate installation in laboratory settings for physical modeling, where various coating materials can be used to ensure there is minimal influence on the model, ensuring the validity of the scaling laws. Real-time data transmission is another advantage, providing timely insights into the structural response and loading conditions of geotechnical structures, even from remote locations. On the other hand, traditional monitoring techniques typically rely on multiple separate sensors, require extensive wiring, and involve complex installation processes, making them less practical for large-scale or remote applications. These techniques do not always provide real-time data transmission or remote monitoring, limiting their effectiveness in dynamic environments. The knowledge gained from the current study can potentially be applied to the use of FBG sensors in other geotechnical models in the laboratory or in the field, utilizing seismic and/or centrifuge tests, including impact scenarios. This may include applications such as foundations, retaining walls, and tunneling projects, where monitoring structural behavior under various loading conditions is critical.

However, while the benefits are clear, there are some limitations and risks that should be taken into consideration. For example, the installation of such sensors in a delicate and historically significant environment requires careful planning to avoid any damage to the structure. Furthermore, as with any advanced technology, the initial costs, specialized equipment, and the complexity of data interpretation could present barriers, particularly in smaller-scale or resource-constrained projects. Ensuring long-term data reliability and system functionality, including periodic maintenance, is important for the sustained effectiveness of these systems. When integrated thoughtfully, FBG sensors can significantly enhance conservation strategies by offering reliable, real-time insights, aiming to preserve the integrity of historical sites.

Future advancements in optical fiber sensor technologies for geotechnical and heritage monitoring could focus on several key areas. One area for development could be improving the durability of sensors, particularly in harsh environmental conditions, such as extreme temperatures, humidity, or exposure to corrosive elements. Advances in protective coatings and materials for the sensors could enhance their lifespan and reliability in challenging monitoring environments. Additionally, there is a need for further research into improving data analysis techniques. As optical fiber sensors generate large volumes of data, the development of more efficient and advanced algorithms for real-time data processing and interpretation is critical. Incorporating machine learning and artificial intelligence could enhance predictive capabilities and enable more effective decision-making in geotechnical and heritage-monitoring applications. Overall, these advancements could lead to more efficient, cost-effective, and accurate monitoring systems for both geotechnical and cultural heritage projects, ensuring better preservation and management.

## Figures and Tables

**Table 1:** Single-axis acceleration sensor specifications.

Specifications	
Sensitivity	75 pm/g @ 40 Hz
Measurement range	±10 g
Frequency range	0 to 50 Hz
Resonance frequency	430 Hz
Flatness	<2%
Resolution	12.5 μg/√Hz
Maximum calib. Error	±0.1 g @ 40 Hz
Transverse sensitivity	<0.1%

## Data Availability

Data are contained within the article.
